# Human Bertiellosis in Sri Lanka’s Dry Zone: A Rare Documented Case Report

**DOI:** 10.7759/cureus.105949

**Published:** 2026-03-26

**Authors:** Chamara Sarathchandra, Kosala Weerakoon, Lanka Wijekoon, Prasanna Weerawansa, Sisira Siribaddana

**Affiliations:** 1 Department of Medicine, Rajarata University of Sri Lanka, Anuradhapura, LKA; 2 Department of Parasitology, Rajarata University of Sri Lanka, Anuradhapura, LKA

**Keywords:** bertiella studeri, one health, sri lanka, tapeworm infection, zoonotic helminthiasis

## Abstract

*Bertiella studeri* is a zoonotic cestode that infects humans through accidental ingestion of cysticercoid-infected oribatid mites, with non-human primates serving as definitive hosts. Human bertiellosis remains rare and underdiagnosed, particularly in regions where primates interact closely with human populations. We report a confirmed case of *B. studeri* infection in a 41-year-old woman from Anuradhapura District in Sri Lanka's dry zone. The patient presented with a six-week history of nonspecific abdominal symptoms, weight loss, and passage of whitish, mobile segments in her stool. Microscopic examination confirmed the presence of characteristic *Bertiella* eggs and proglottids; however, molecular confirmation was not performed due to the unavailability of appropriate testing facilities. She was successfully treated with praziquantel, resulting in complete symptom resolution and parasite clearance. This case expands the known geographic distribution of human bertiellosis in Sri Lanka beyond the previously reported wet zone and highlights diagnostic challenges in resource-limited settings. The case highlights One Health concerns regarding increasing human-primate contact, limited access to anti-cestodal medications, and the need for improved environmental hygiene in endemic areas.

## Introduction

*Bertiella* is a genus of small intestinal cestodes (anoplocephalid tapeworm) that primarily infects non-human primates [1]. Two species within this genus, *Bertiella studer*i and *B. mucronata*, cause human infection. While *B. mucronata* is predominantly reported in South America and the Caribbean, *B. studeri* has been documented in Africa and Asia [1,2]. The life cycle involves non-human primates (genera *Macaca*, *Cercopithecus*, and *Semnopithecus*) as definitive hosts and free-living oribatid soil mites as intermediate hosts [3]. Within these mites, the parasite develops into the infective cysticercoid stage [1]. Unlike common cestode infections such as *Taenia* and *Hymenolepis* species, *Bertiella* infection is characteristically distinguished by the passage of motile proglottids or tapeworm segments in stool, the absence of hooks on the scolex (unarmed scolex), and its unique epidemiological link to non-human primate habitats and oribatid mite exposure [1].

Human transmission occurs through accidental ingestion of infected mites via contaminated soil, food, or water [1]. Once ingested, the larvae develop into adult tapeworms within the gastrointestinal tract [1]. In Sri Lanka, the endemic toque macaque (*Macaca sinica*) and grey langur (*Semnopithecus priam*) have adapted to human-dominated environments, establishing a significant zoonotic interface [4]. The first reported case of human *Bertiella* infection in Sri Lanka occurred in 1976 [5]. Since then, a few cases have been documented [6-11].

This report describes the first adult case from Anuradhapura District in the North Central Province, in the dry zone of Sri Lanka, representing a notable geographic expansion of reported bertiellosis in Sri Lanka.

## Case presentation

A 41-year-old woman from a rural farming community in Anuradhapura presented with a six-week history of vague, non-radiating abdominal pain localised predominantly to the upper and central abdomen. Associated symptoms included anorexia and an unintentional 2 kg weight loss. She denied fever, vomiting, diarrhoea, or other systemic symptoms. During this period, she reported passing whitish, mobile, flat, square-shaped pieces in her faeces on five occasions, which she initially dismissed as insignificant.

Her past medical history was unremarkable except for a recent episode of right lower limb cellulitis. She worked as a cattle farmer, managing approximately 30 cows and performing daily milking and enclosure cleaning. Although she did not keep pets, monkeys frequently visited her residence in search of food. She consumed a non-vegetarian diet and drank filtered tap water.

On examination, the patient appeared well-nourished and afebrile with a body mass index of 23.2 kg/m². Conjunctivae were pink and anicteric. No lymphadenopathy was detected. Vital signs were stable: pulse rate of 84 beats per minute and blood pressure of 130/80 mm Hg. Systematic examination of the abdominal, cardiovascular, respiratory, and neurological systems revealed no abnormalities.

Laboratory investigations showed a WBC count of 10.1 × 10⁹/L (normal 4.0-11.0), haemoglobin of 10.8 g/dL (normal 11-14), and platelet count of 152 × 10⁹/L (normal 150-400). C-reactive protein was 2 mg/L (normal <6), and serum creatinine was 84 µmol/L. An abdominal ultrasound scan was unremarkable except for a grade 1 fatty liver.

**Figure 1:**
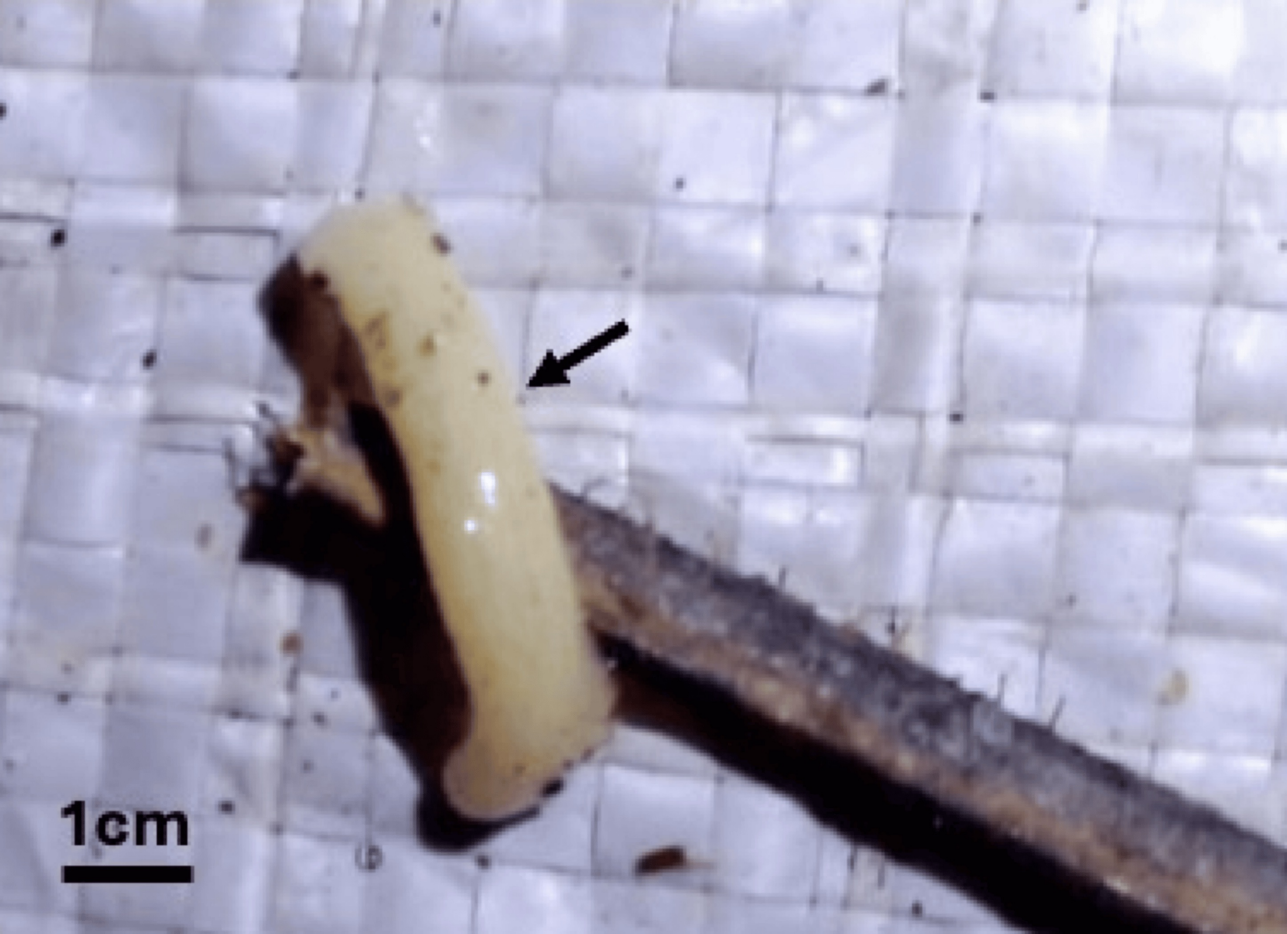
Segment of Bertiella proglottids recovered from the patient's stool sample. The arrow indicates the proglottid freshly passed in stool.

**Figure 2:**
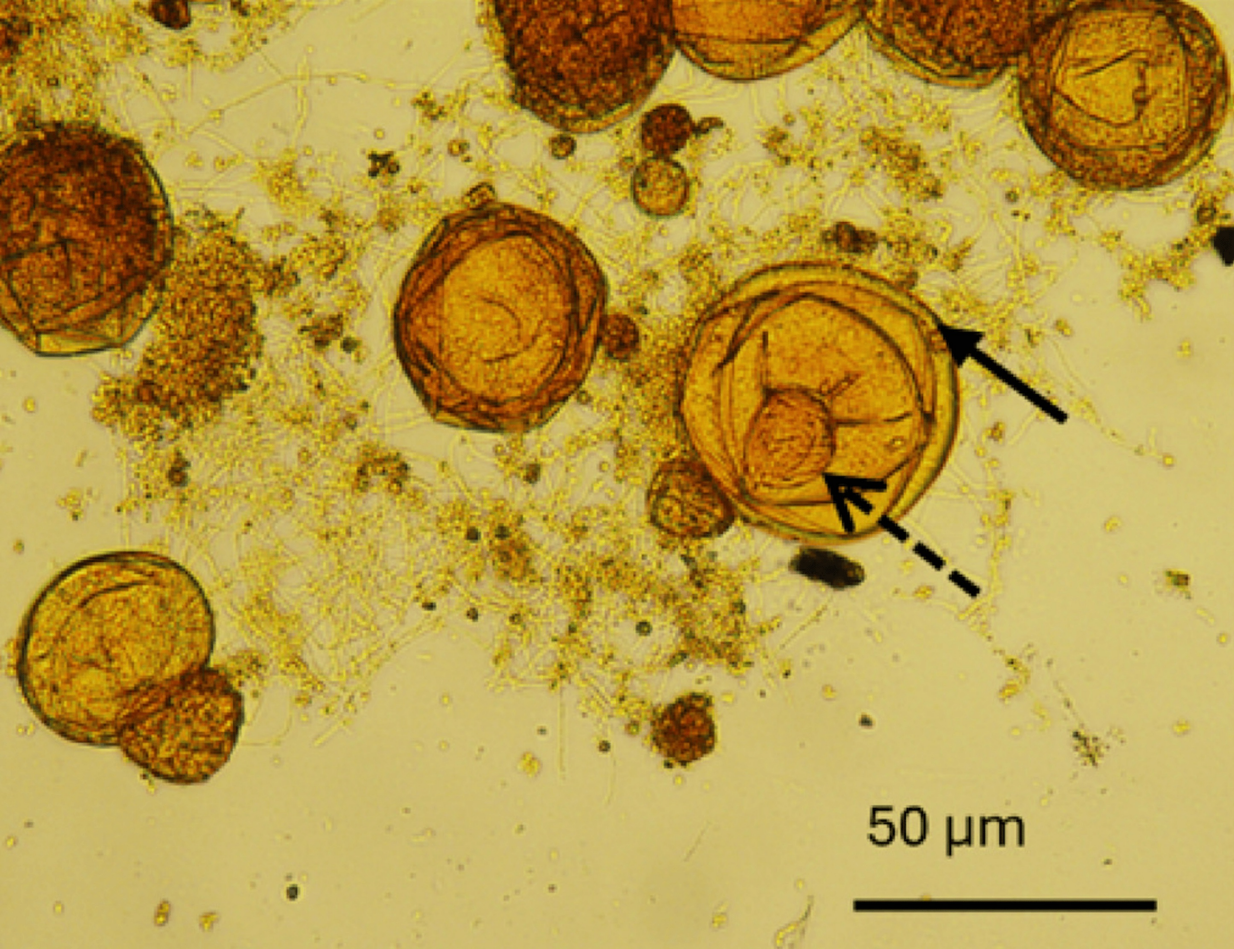
Iodine-stained, microscopic view of eggs of Bertiella recovered from the proglottids. Solid arrow: rough non-striated outer membrane, dashed arrow: oncosphere

Macroscopic examination of the whitish bodies retrieved from the patient's faeces revealed proglottids consistent with a tapeworm, suspected to be* B. studeri*. Microscopic examination of proglottid extracts demonstrated characteristic spherical eggs (40-50 μm in diameter) containing hexacanth embryos with the distinctive pyriform apparatus, confirming the diagnosis of *B. studeri* infection (Figures 1, 2). However, molecular confirmation could not be performed due to the unavailability of appropriate diagnostic facilities. In addition, multiple stool samples were examined using direct wet mount microscopy and the formalin-ether sedimentation concentration technique, which did not demonstrate evidence of other parasitic infections.

The patient was treated with a single dose of 600 mg of oral praziquantel. At one-month follow-up, she reported complete resolution of abdominal symptoms and cessation of proglottid passage. Repeat stool examination at one month's follow-up showed no eggs or proglottids, confirming successful parasite eradication.

## Discussion

**Figure 3:**
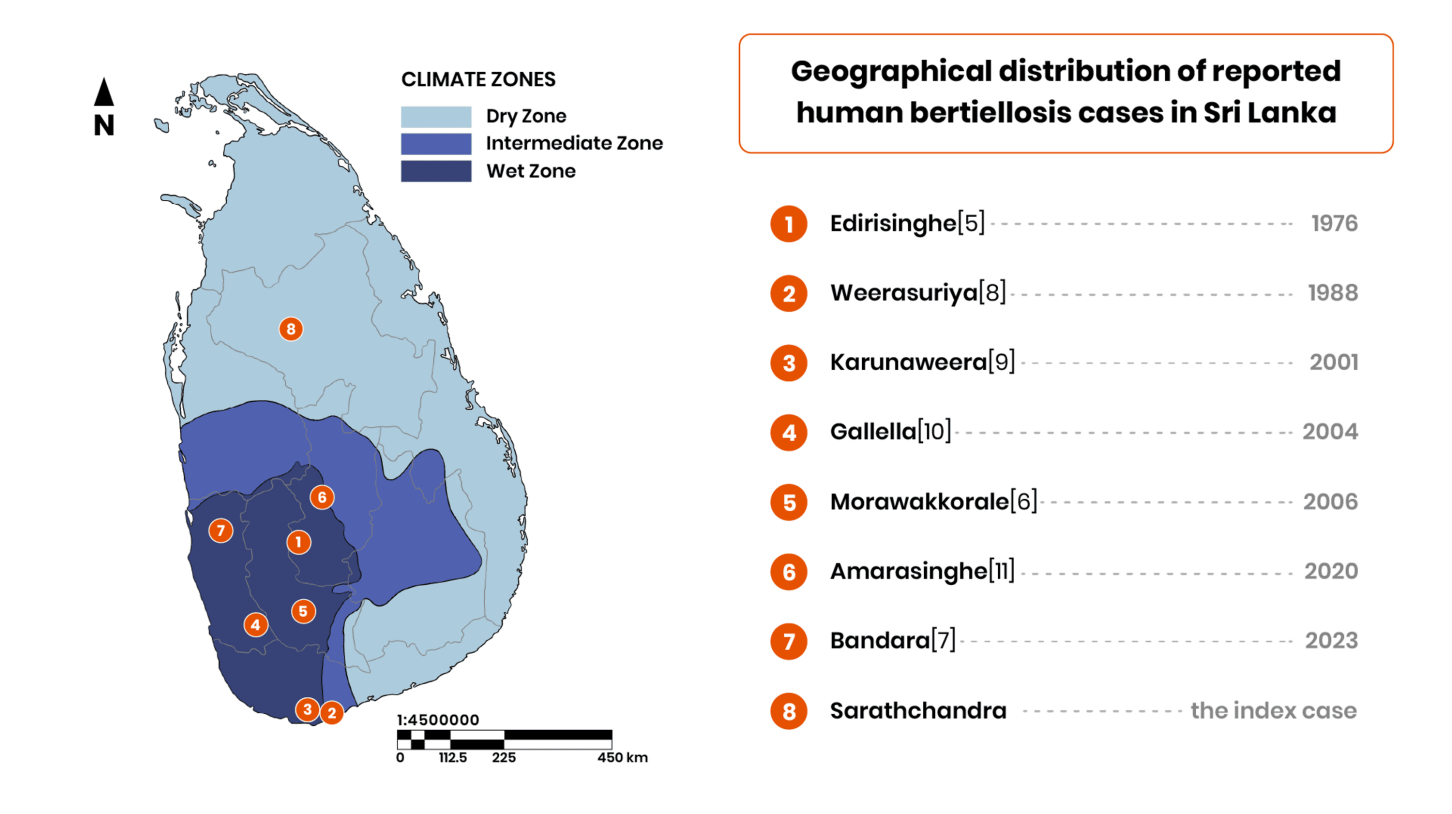
Geographical distribution of reported human bartiellosis cases in Sri Lanka. Geographical coordinates obtained from the Department of Meteorology (https://meteo.gov.lk)/ were utilised to delineate and classify the boundaries of the climate zones. Numbered markers indicate reported case locations; superscript numbers correspond to published references. Shaded areas represent climatic zones (dry, intermediate, and wet zones).

This case represents the first documented instance of adult human bertiellosis in Anuradhapura District and, notably, the first case reported from Sri Lanka's dry zone. All previously documented cases occurred in the wet zone, encompassing the Western, Southern, Sabaragamuwa, and Central Provinces (Table 1, Figure 3). This geographic distribution pattern likely reflects the ecology of intermediate hosts: oribatid mites preferentially inhabit cool, moist soil environments [12].

**Table 1:** Summary of human bertiellosis cases reported from Sri Lanka

Publication	Province (city, village)	Demography	Symptoms and findings	Diagnostic method	Treatment	Outcome
Edirisinghe^5^ 1976	Central (Nawalapitiya)	4.5-year-old male	Passage of proglottids	Not reported	Niclosamide	Not reported
Weerasuriya^8^ 1988	Southern (Dickwella)	2-year-old male (two children)	Passage of proglottids	Morphological	Niclosamide	Successful in case one, no information on the other child
Karunaweera^9^ 2001	Southern (Dickwella)	10 half-year-old female	Bleeding per rectum; passage of proglottids	Morphological	Niclosamide, bisacodyl	Successful
Gallella^10^ 2004	Western (Kaluthara Baduraliya)	30-month-old male	Passage of proglottids	Morphological	Niclosamide 3 times, then praziquantel with polyethylene glycol	Niclosamide resistance, successful treatment with Praziquantel
Morawakkorale^6^ 2006	Sabaragamuwa (Kahawatte)	30-month-old male	Passage of proglottids	Morphological	Niclosamide, bisacodyl	Successful
	Sabaragamuwa (Nivithigala)	5-year-old male	Passage of proglottids	Morphological	Niclosamide and then praziquantel and bisacodyl	Successful
Amarasinghe^11^ 2020	Central	24 patients, 3.5-9 years	Passage of proglottids; some had abdominal pain and diarrhea.	This study provides the molecular analysis of the B. studeri tapeworms. They have used proglottids from 24 children.	Not reported	Not reported
Bandara^7^ 2023	Western (Imbulgoda Gampaha)	3-year-old female	Loose stools, abdominal pain, reduced appetite for 6 months	Morphological	Praziquantel and bisacodyl	Not reported

Human bertiellosis cases typically involve individuals with close environmental or occupational contact with non-human primates, the definitive hosts [1]. In this case, the patient's residence in an area frequented by monkeys suggests a plausible transmission route. Her occupation as a cattle farmer, involving outdoor work and potential soil exposure, further increased her risk of ingesting infected mites.

The emergence of this case from the dry zone raises important epidemiologic questions. Climate variability, changing land use patterns, and altered human-wildlife interactions may be expanding the habitat suitability for oribatid mites or increasing transmission opportunities. Alternatively, improved diagnostic awareness may reveal previously unrecognised endemic foci.

The clinical spectrum of bertiellosis ranges from asymptomatic to nonspecific gastrointestinal symptoms, including abdominal pain, diarrhoea, anorexia, and weight loss [13-16]. Our patient presented with mild but persistent symptoms. The passage of visible proglottids provided a crucial diagnostic clue, although many patients may not recognise or report this finding.

Definitive diagnosis relies on the identification of eggs and proglottids in stool samples. *B. studeri* eggs are spherical with thick shells, featuring a rough non-striated outer membrane, and contain a characteristic hexacanth embryo (pyriform apparatus) [14]. These morphologic features distinguish *Bertiella* from other cestode species. This case highlights the importance of maintaining such diagnostic expertise in endemic regions, particularly where laboratory resources may be limited [1,17].

Praziquantel remains the treatment of choice for bertiellosis, acting through calcium channel disruption to paralyse and facilitate expulsion of the adult worm [1,17]. Niclosamide represents an alternative agent [15], although resistance has been reported in Sri Lanka [6,7]. A critical public health concern is that neither praziquantel nor niclosamide currently appears on Sri Lanka's essential medicines list, creating significant access barriers for patients with cestode infections [6,7]. This gap in essential drug availability warrants urgent attention from health authorities.

This case illustrates important One Health dimensions at the human-animal-environment interface. Increasing human coexistence with non-human primates, combined with inadequate waste management and poor soil sanitation, perpetuates zoonotic transmission cycles [3]. Comprehensive public health interventions should include environmental sanitation improvements, community education about transmission risks, and evidence-based wildlife management strategies [18].

Although this is the first documented case from Anuradhapura District, in the dry zone, the actual prevalence of *Bertiella* infection may be underestimated due to underdiagnosis and underreporting [1]. The widespread presence of intermediate hosts (oribatid mites) and the abundant definitive host populations support the likelihood of additional undetected or asymptomatic infections [1,13]. Enhanced surveillance and increased clinical awareness are needed to better define the true burden of bertiellosis in Sri Lanka.

## Conclusions

This case documents the first adult bertiellosis case from Anuradhapura District and extends the known geographic distribution of human bertiellosis in Sri Lanka to include the dry zone (Figure 3). The successful diagnosis and treatment demonstrate that bertiellosis can be identified and managed effectively in resource-limited settings, provided parasitologic expertise is maintained. Notably, the absence of praziquantel from Sri Lanka's essential medicines list posed a practical treatment access barrier in this case, a gap warranting attention from health authorities. Whether this case reflects a true geographic expansion of transmission, enhanced diagnostic recognition, or a previously undetected endemic focus remains to be determined through systematic surveillance. From a One Health perspective, these findings suggest the need for further investigation into human-primate interfaces and environmental risk factors - though conclusions about the broader epidemiologic scope and public health burden of bertiellosis in Sri Lanka await evidence from larger, population-based studies.
